# Supplementary material for: Clinically relevant treatment of PDX models reveals patterns of neuroblastoma chemoresistance
Source: Sci Adv. 2022 Oct 28;8(43):eabq4617. doi: 10.1126/sciadv.abq4617 (PMC9616506; doi:10.1126/sciadv.abq4617)

Science Advances
AAAS

Supplementary Materials for

Clinically relevant treatment of PDX models reveals patterns of neuroblastoma chemoresistance

Adriana Mañas *et al.*

Corresponding author: Daniel Bexell, daniel.bexell@med.lu.se

Sci. Adv. **8**, eabq4617 (2022)
DOI: 10.1126/sciadv.abq4617

The PDF file includes:

Figs. S1 to S10
Tables S1 and S2
Legends for data files S1 to S4

Other Supplementary Material for this manuscript includes the following:

Data files S1 to S4

Supplementary Figure 1

A

	Tumor Type	Tumor Site	Stage	Diff. Status	Treatment Before Collection	Patient COJEC Response	Cause of Death	Main Copy Number Aberrations
**PDX1**	Primary	Adrenal Gland	IV	Undiff.	No	Refractory	Primary Tumor	*MYCN* Amp, 1p-, 17q+
**PDX2**	Metastasis	Brain	IV	Undiff.	Yes	Responsive	Relapse	*MYCN* Amp, 1p-, 17q+
**PDX3**	Primary	Adrenal Gland	III	Poorly Diff.	No	Responsive	Relapse	*MYCN* Amp, 1p-, 17q+

B

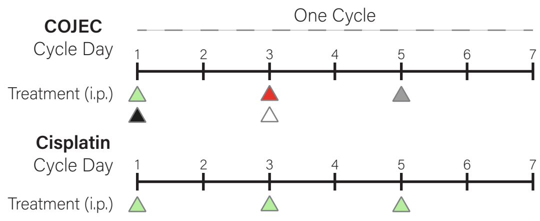

	Basic Dose (mg/kg)	High Dose (mg/kg)
Cisplatin	1	1.5
Vincristine	0.25	0.5
Etoposide	4	6
Cyclophosphamide	75	100
Carboplatin	25	35
Cisplatin	4	

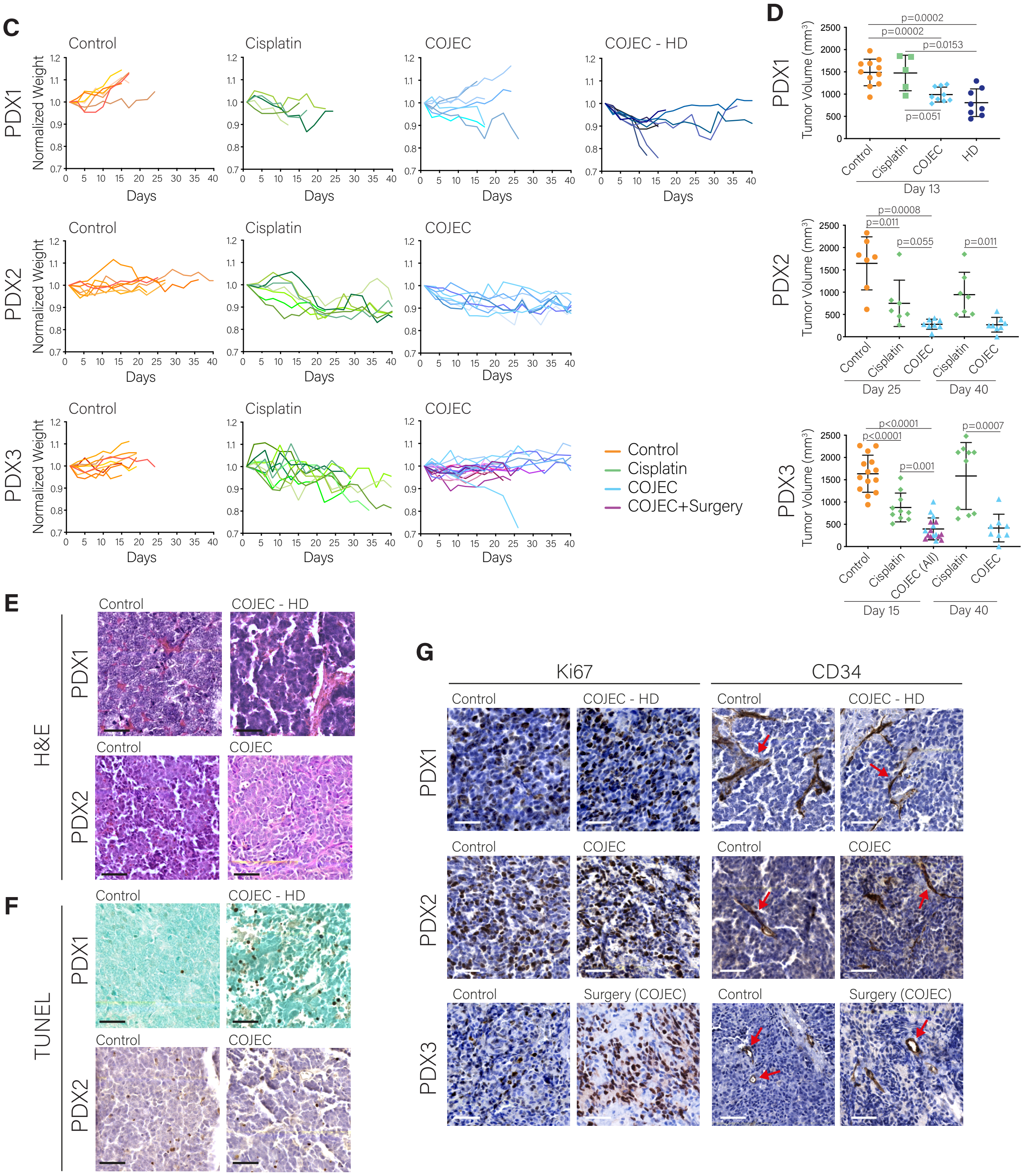

Fig. S1. A COJEC-like protocol for the treatment of NB PDX models *in vivo*. (**A**) Summary of general characteristics of the NB PDX models. (**B**) Dosing schedule for the COJEC-like cycle and the cisplatin cycle, and drugs doses for basic and high dose tiers. i.p., intraperitoneal. (**C**) Normalized mouse weight during treatment. Mice received a maximum of 6 cycles of treatment. (**D**) Tumor volume comparison across treatment groups at the relevant survival days (PDX1, day 13; PDX2, day 25; and PDX3, day 15) and at the end of treatment (day 40). The last available point was considered when mice had been euthanized before the day of comparison. Significance was determined by ordinary one-way ANOVA with Welch's t-test correction for multiple comparison. (**E**) Hematoxylin and eosin (H&E) staining of PDX1 and PDX2 to assess morphological differentiation. (**F**) TUNEL staining of PDX1 and PDX2 to assess cell death. (**G**) Ki67 (proliferation marker) and CD34 (blood vessel marker) immunohistochemical staining for each PDX model. Red arrows indicate representative blood vessels. Scale bars, 50 μm.

Supplementary Figure 2

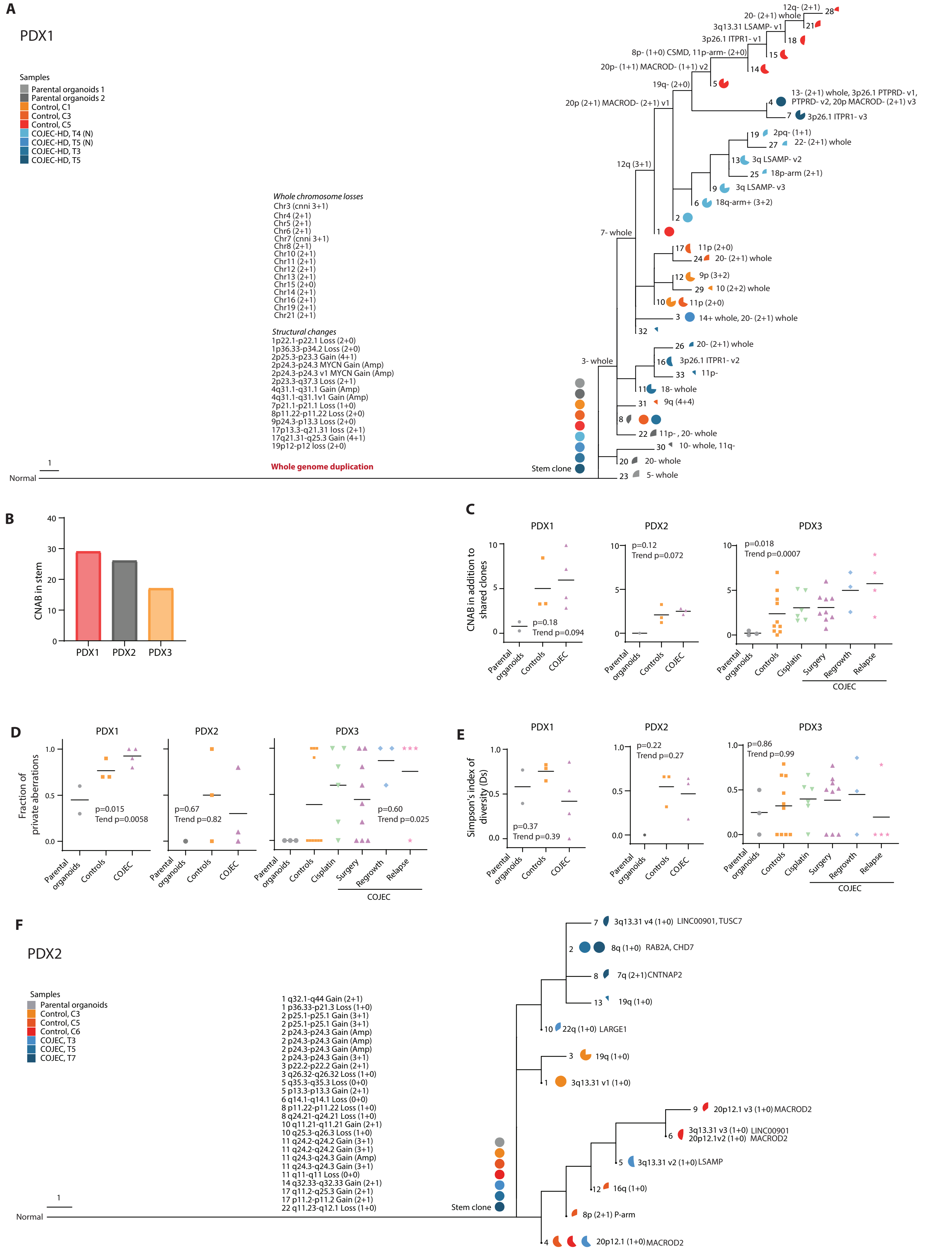

Fig. S2. Clonal dynamics (PDX1 and 2) and genetic diversity across NB PDX models. (**A**) Maximum-likelihood (ML) phylogenetic tree of PDX1 based on CNAs. Frequency of different subclones is visualized with circle diagrams. Whole-genome duplication (WGD) early in the stem of PDX1 was followed by a high number of large CNA events where the majority involved loss of the fourth copy of a region. Multiple occasions of parallel evolution were detected in PDX1. (**B**) Number of CNAs in phylogenetic stem of each PDX model. (**C**) Copy number aberration burden (CNAB), (**D**) the fraction of private aberrations and (**E**) genetic diversity (Ds) per treatment group in PDX1, 2, and 3. Comparison between all groups in each graph and test for trend by one-way ANOVA. (**F**) ML phylogenetic tree of PDX2. Structural changes in the stem clone are listed.

Supplementary Figure 3

A PDX3 - Maximum likelihood phylogeny

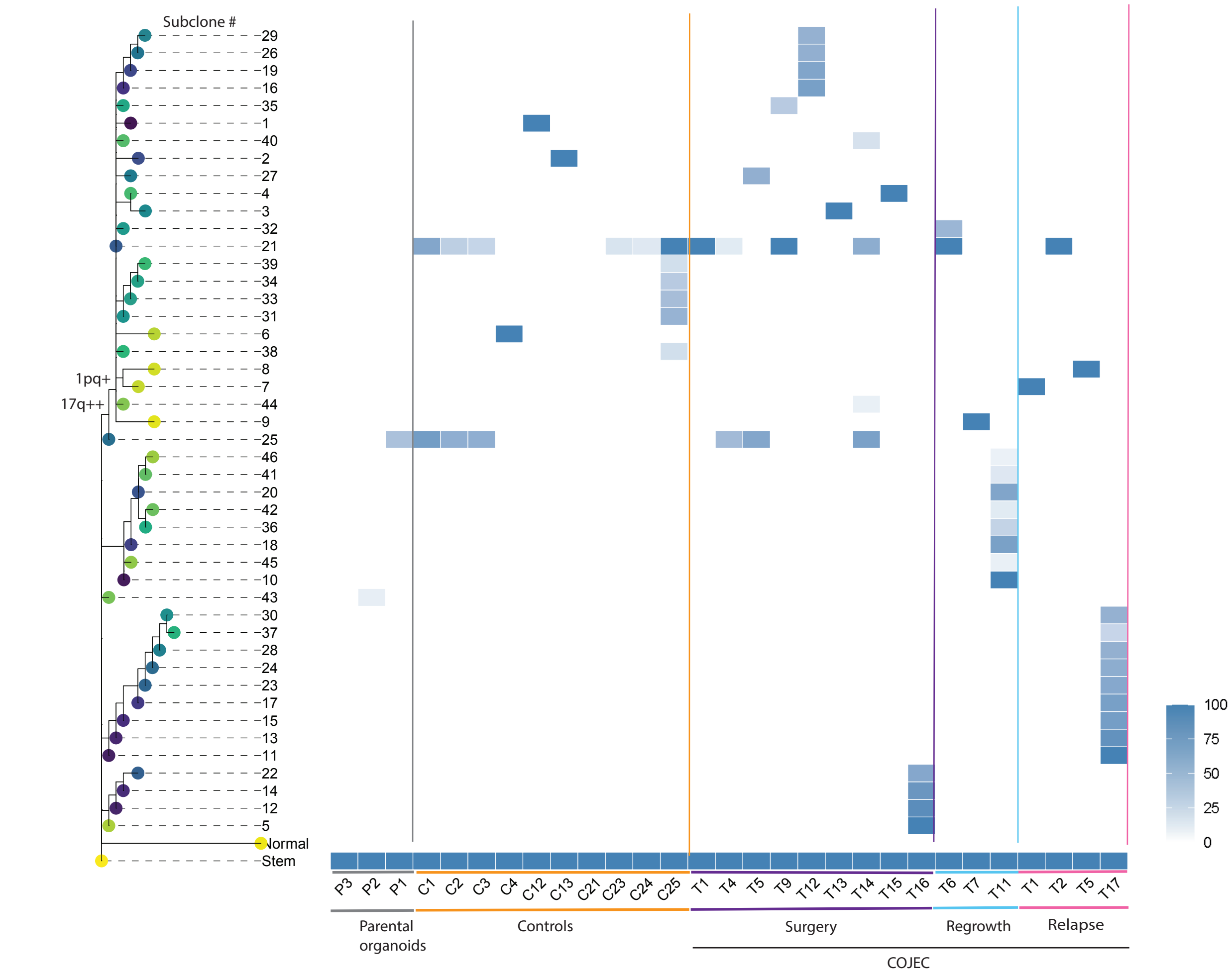

B PDX3 - Maximum likelihood event matrix

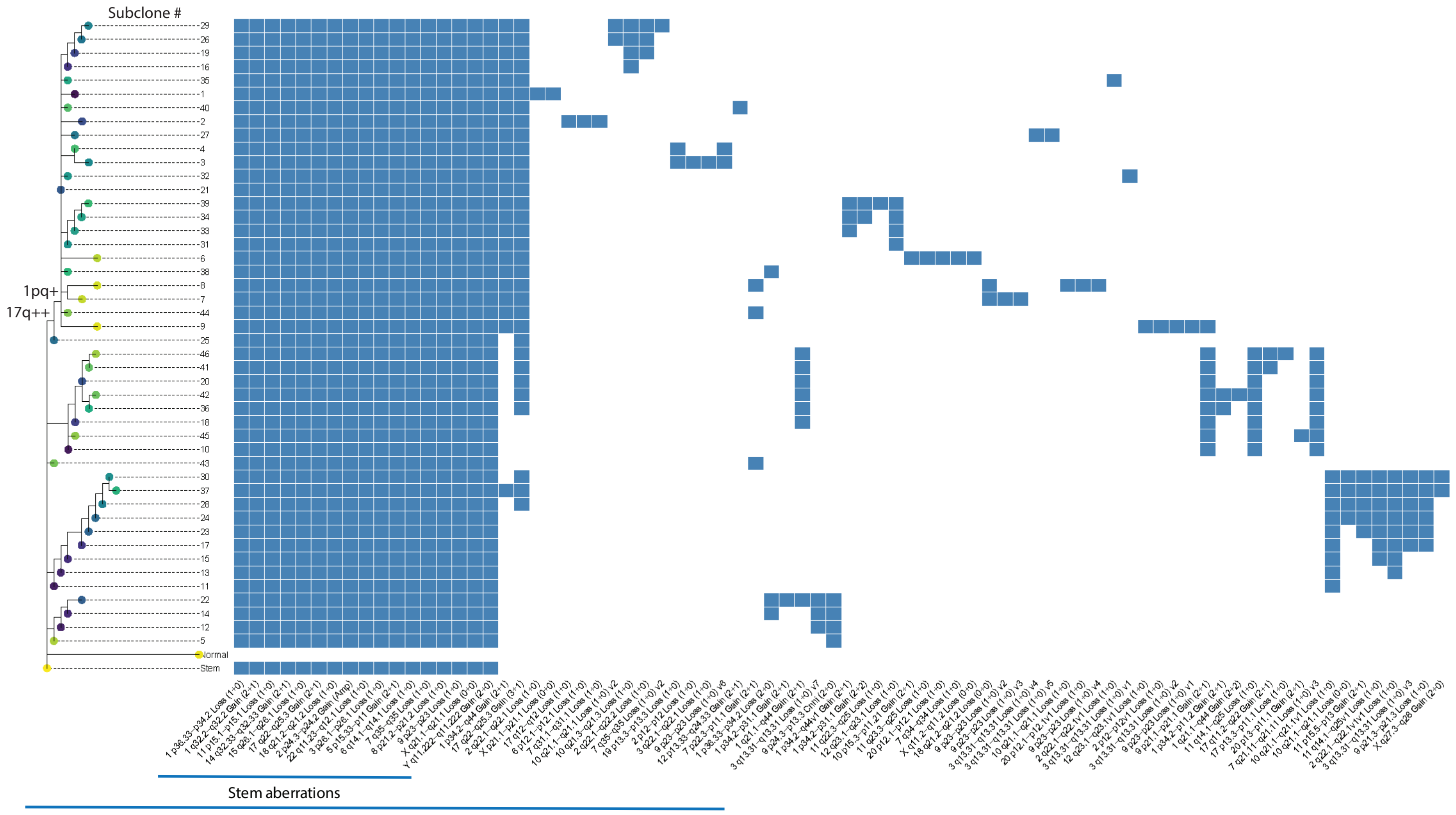

1 p36.33-p34.2 Loss (1+0)
1 q32.2-q32.2 Gain (2+1)
2 p24.3-p24.2 Gain (Amp)
3 p26.1-p26.1 Loss (1+0) (ITPR1)
5 p15.33-p11 Gain (2+1)
6 q14.1-q14.1 Loss (1+0)
7 q35-q35 Loss (1+0)
8 p21.2-p21.2 Loss (1+0)
9p23-p23 Loss (1+0) (PTPRD)
11 p15.1-p15.1 Loss (1+0)
14 q32.33-q32.33 Gain (2+1)
15 q26.1-q26.1 Loss (1+0)
17 q22-q25.3 Gain (2+1)
18 q21.2-q21.2 Loss (1+0)
22 q11.23-q12.1 Loss (1+0)
X q21.1-q21.1 Loss (0+0)
Y q11.222-q11.222 Gain (2+0)

Fig. S3. Heatmap representation of clonal dynamics in PDX3. CNA-based ML phylogenetic trees displaying all clones and subclones detected within PDX3. The trees in A and B are identical. The stem clone is defined as having all CNAs present in all organoids and tumors (lowest row in both trees). Subclones #1 – 46 are present in one or more tumors. The subclone number (#) corresponds to that in Fig. 2. Major branching events, 17q++ and 1pq+, are displayed in the trees. (**A**) Heatmap showing the fraction of each subclone in the respective tumor by the shade of blue. (**B**) Event matrix detailing the genomic location of the respective CNA in each subclone. The CNAs present in the stem are also written in larger text below the tree. The CNAs are presented as chromosome number, position range, type of aberration, copy number, and gene exhibiting convergent evolution, if applicable.

Supplementary Figure 4

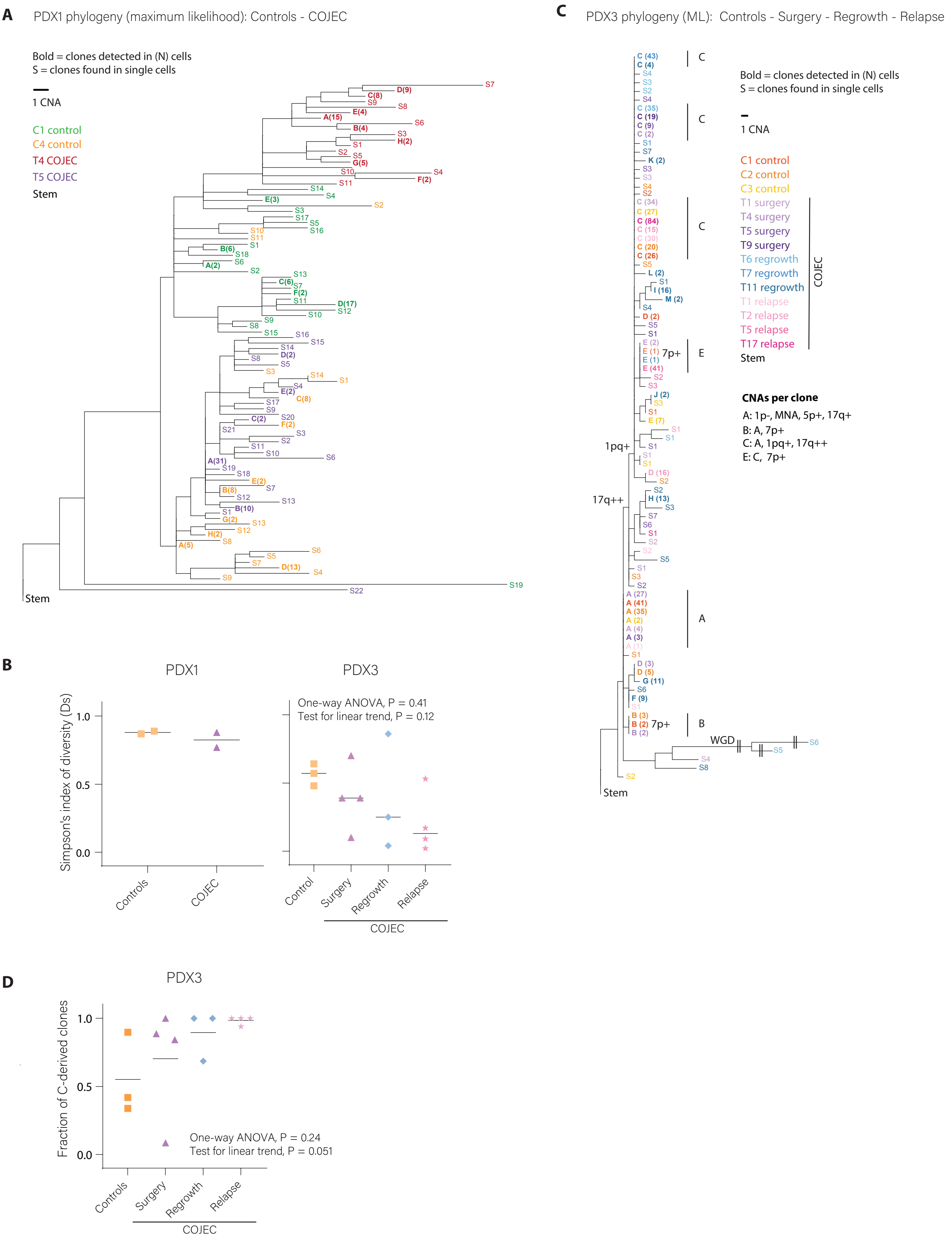

Fig. S4. Phylogenetic analysis of low pass, single-cell (sc) DNA profiles across NB PDX models. (**A**) ML phylogenetic analysis of NB-PDX1 scDNA. Clones labeled with "S" and a number represent single-cell clones that have a unique CNA profile from any other cell among the PDX tumor. Clones represented by multiple cells are indicated by bold letters with the number of cells detected indicated in parentheses. The clone text color reflects the PDX tumor (C1, C4, T4, T5) in which the clone was detected. All clones are unique to a single PDX1 tumor. (**B**) Genetic diversity (Ds) in tumors for the indicated treatment groups of PDX1 and PDX3. Comparison between groups and test for trend in PDX3 with one-way ANOVA. (**C**) ML phylogeny of PDX3 scDNA. Major selective sweeps of 17q++ and 1pq+ are marked. In PDX3, identical clones [A, B, C, E] were found in several of the tumors. (**D**) Fraction of clones derived from C-clone in the different PDX3 treatment groups. Comparison between groups and test for trend with one-way ANOVA

Supplementary Figure 5

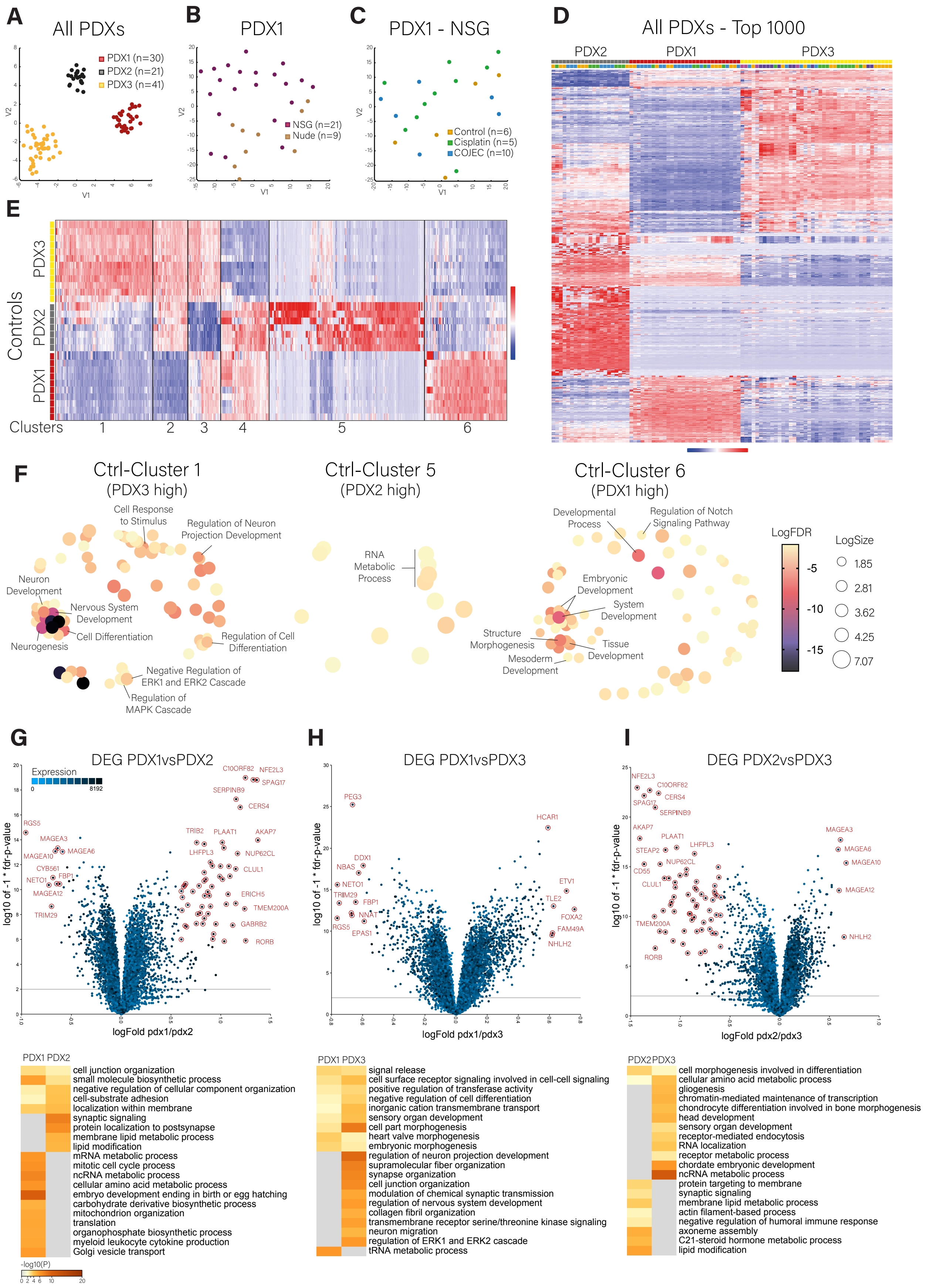

Fig. S5. Baseline transcriptional signatures for each NB PDX model. (**A-C**) t-SNE plots of all PDXs colored by PDX model (A), PDX1 colored by mouse strain (B), PDX1 (NSG mice only) colored by treatment group (C). (**D**) Unsupervised top 1000 differentially expressed genes for all samples. (**E**) Unsupervised top 1000 differentially expressed genes for control samples. Six gene clusters are identified. (**F**) Multidimensional scaling (MDS) analysis and visualization of gene ontologies defined by Clusters 1, 5 and 6 from control (Ctrl) PDX samples in (E) (Revigo, non-redundant scaling 0.7). (**G-I**) Analysis of differentially expressed genes (DEG) between control samples of each PDX. Top panels display volcano plots of all DEG for each paired comparison (red genes = FDR < 0.01 and |fold change| >1.5, R2 Genomics Analysis Platform). Bottom panels display gene ontologies defined by the top 1000 DEG for each paired comparison (Metascape enrichment analysis for GO Biological processes).

Supplementary Figure 6

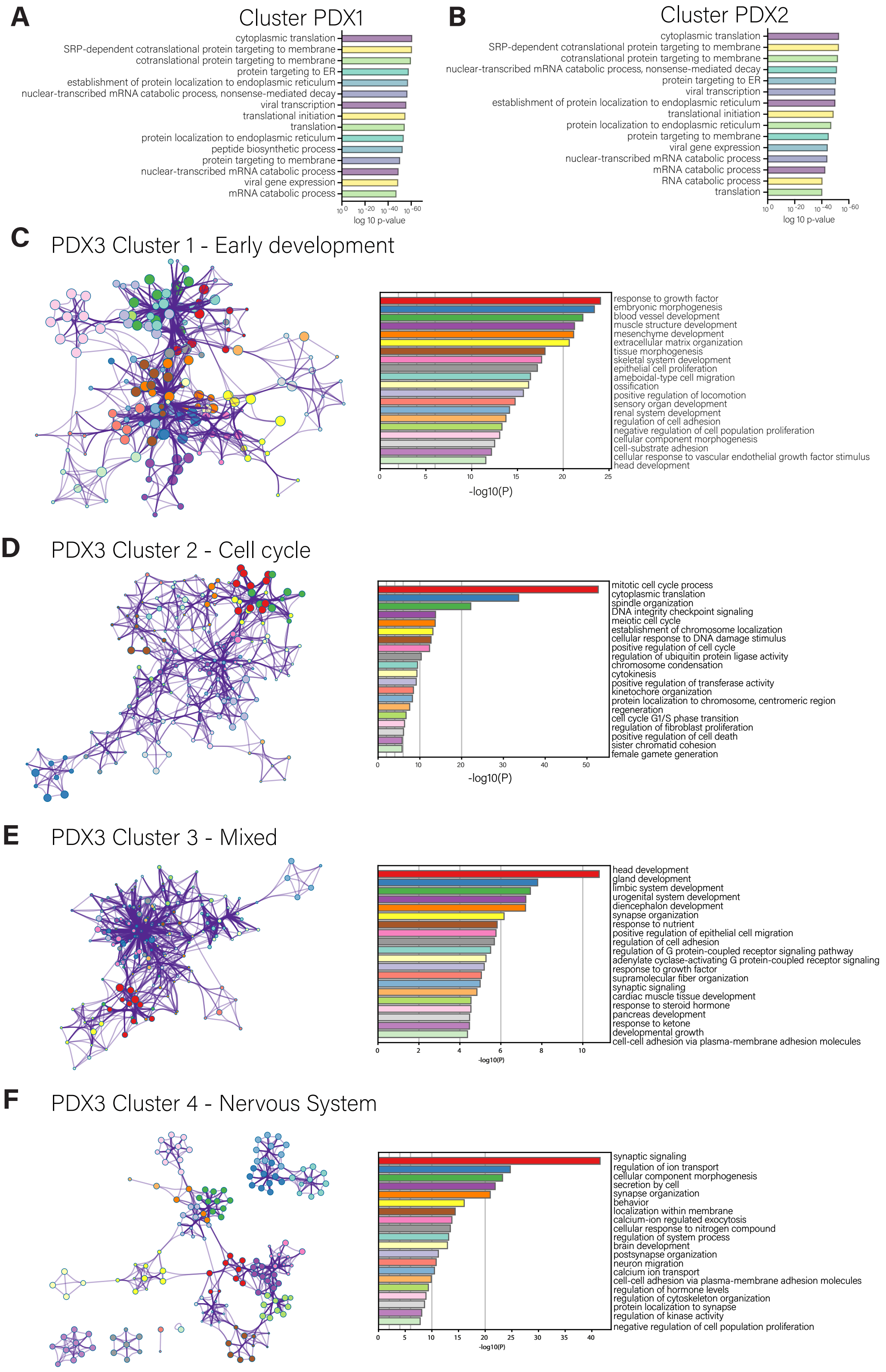

Fig. S6. Characterization of transcriptional signature clusters for each NB PDX. (**A-B**) Top 15 most significant gene ontologies defined by the single clusters identified for PDX1 and PDX2 (see Fig. 4E-F). (**C-F**) Enrichment analysis for the 4 clusters defined by PDX3 (see Fig. 4G), visualized through network plots based on the top 20 most significant ontology clusters (Metascape enrichment analysis for GO Biological processes).

Supplementary Figure 7

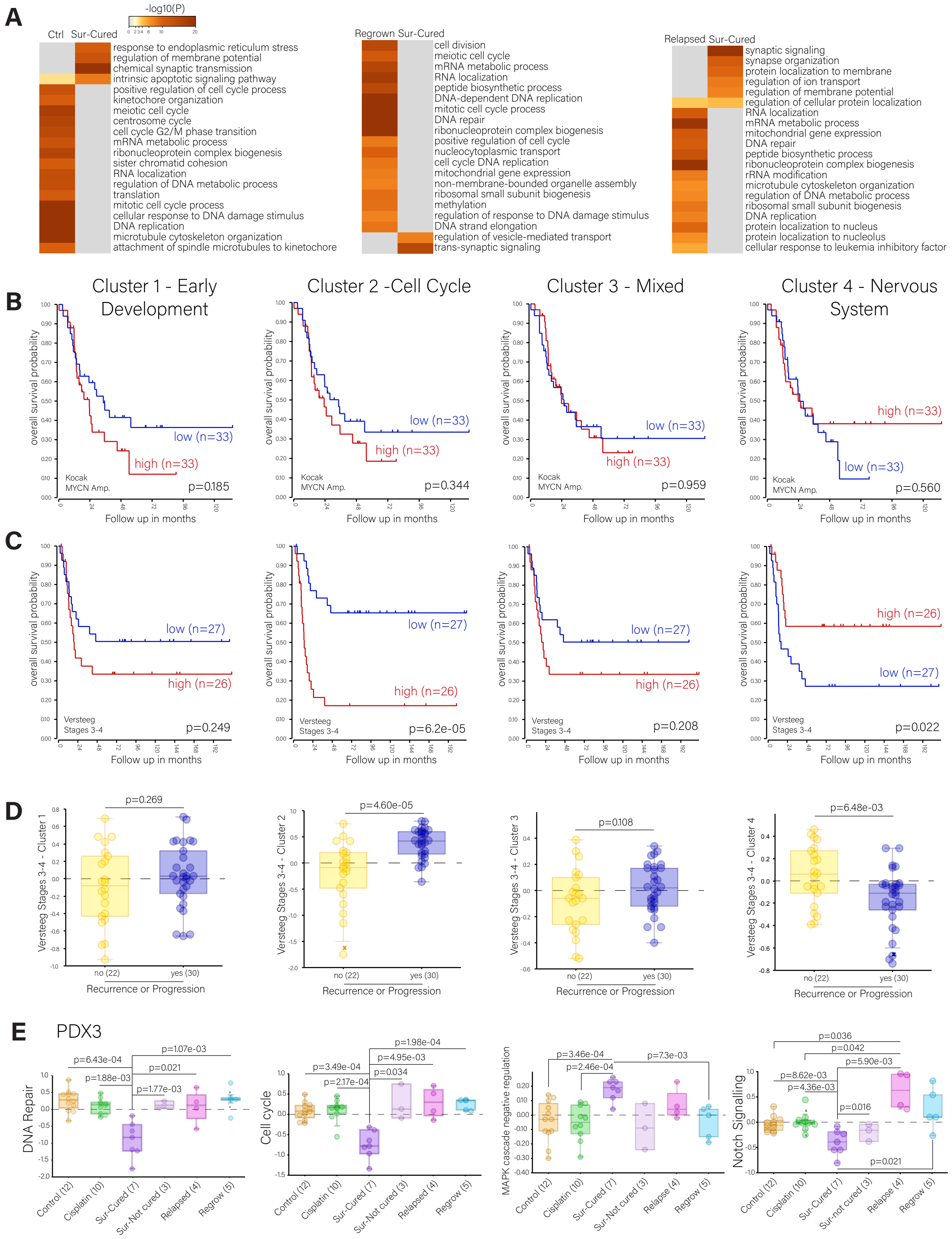

Fig. S7. Treatment response signatures across PDX3 treatment groups and NB patient datasets. (**A**) Gene ontologies defined by the top 1000 DEG between PDX3 treatment groups (Metascape enrichment analysis for GO Biological processes). (**B, C**) Kaplan-Meier overall survival curves for each PDX3 gene cluster identified from Fig. 4G in NB patients. *MYCN* amplified tumors from Kocak dataset (B) and stages 3-4 patients from Versteeg dataset (C). R2 Genomics Analysis Platform, median cut, log-rank test. (D) Comparison of the expression (average z-score values over the gene set) of each PDX3 gene cluster identified from Fig. 4G, between patients that presented (yes) or not (no) a recurrence and/or tumor progression under treatment (Versteeg dataset). (**E**) Statistical analysis with ordinary one-way ANOVA and Welch's t-test correction for multiple comparison of DNA repair, Cell cycle, MAPK cascade negative regulation, and Notch signaling, across PDX3 treatment groups.

Supplementary Figure 8

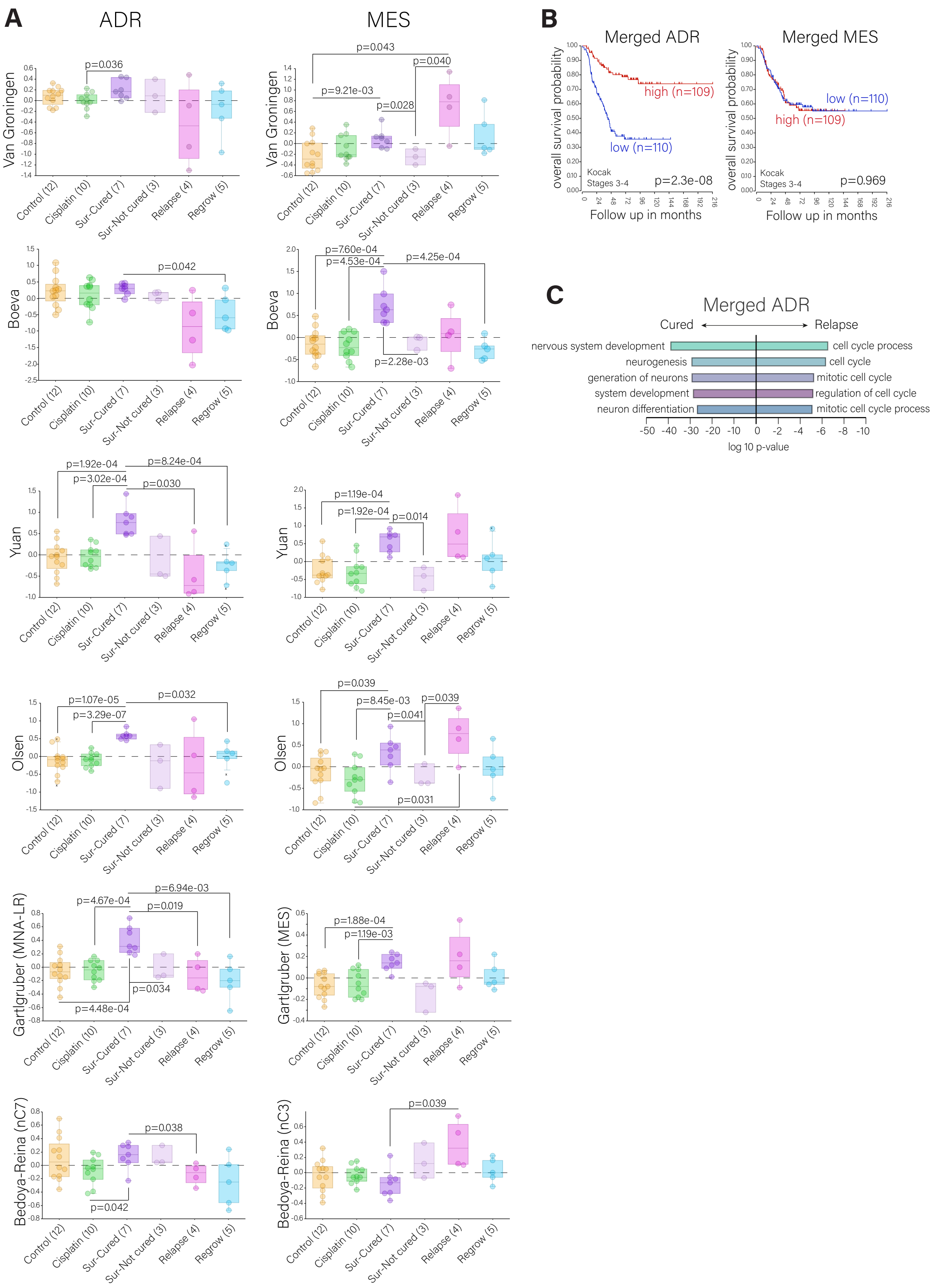

Fig. S8. Publicly available ADR and MES-like gene signatures across PDX3 treatment groups. (**A**) Analysis of six publicly available pairs of ADR-MES gene expression signatures *(10, 11, 15–18)* across PDX3 treatment groups. (B) Kaplan-Meier overall survival curves for the Merged ADR and MES gene signatures in high-risk NB patients (stages 3-4, Kocak dataset, R2 Genomics Analysis Platform). Statistical analysis with log-rank test. (**C**) Bar graph presenting the top 5 gene ontologies derived from the ADR-cured and ADR-Relapse sub-signatures.

Supplementary Figure 9

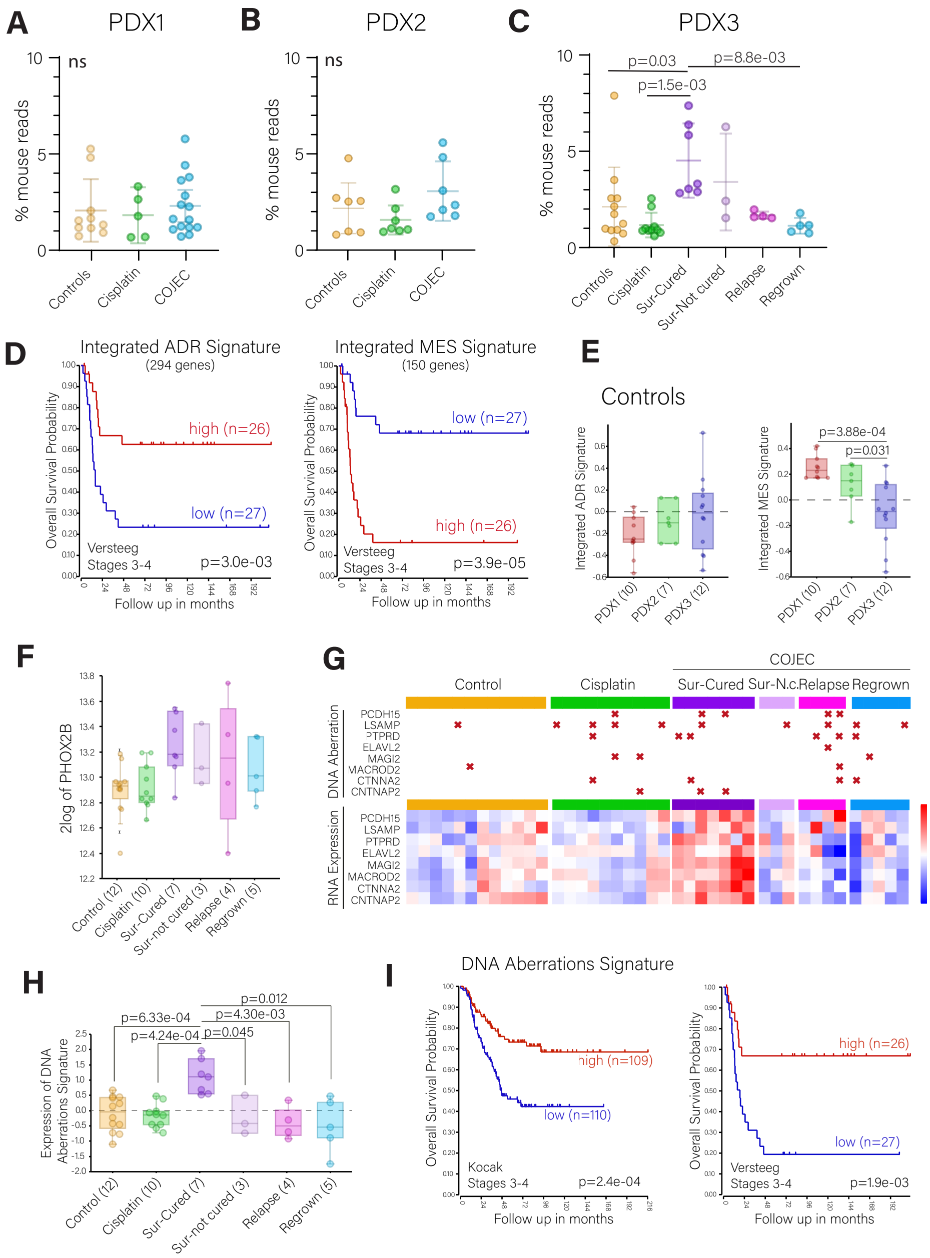

Fig. S9. Human vs. mouse RNA transcripts, and correlation of the Integrated ADR/MES signatures and key genetic aberrations with NB patient prognosis. (**A-C**) Percentage of mouse RNA reads in each tumor sample across NB PDXs. ns, non-significant. (**D)** Kaplan-Meier overall survival curves for the Integrated ADR and Integrated MES gene signatures in high-risk NB patients (stages 3-4, Versteeg dataset, R2 Genomics Analysis Platform). Statistical analysis with log-rank test. **(E**) Expression (average z-score values over the gene set) of the Integrated ADR and MES signatures across the control samples of the three PDX models. Statistical analysis performed with R2 genomics platform with ordinary one-way ANOVA and Welch's t-test correction. (**F**) Gene expression levels of *PHOX2B* across PDX3 treatment groups. (**G**) Comparison between convergent DNA aberrations (losses) and RNA expression of the corresponding genes for each PDX3 sample. (**H**) Statistical analysis of the signature from (G) across PDX3 treatment groups. (**I**) Kaplan-Meier overall survival curves for the signature from (G) for Stage 3-4 patients from the Kocak and Versteeg datasets.

Suplementary Figure 10

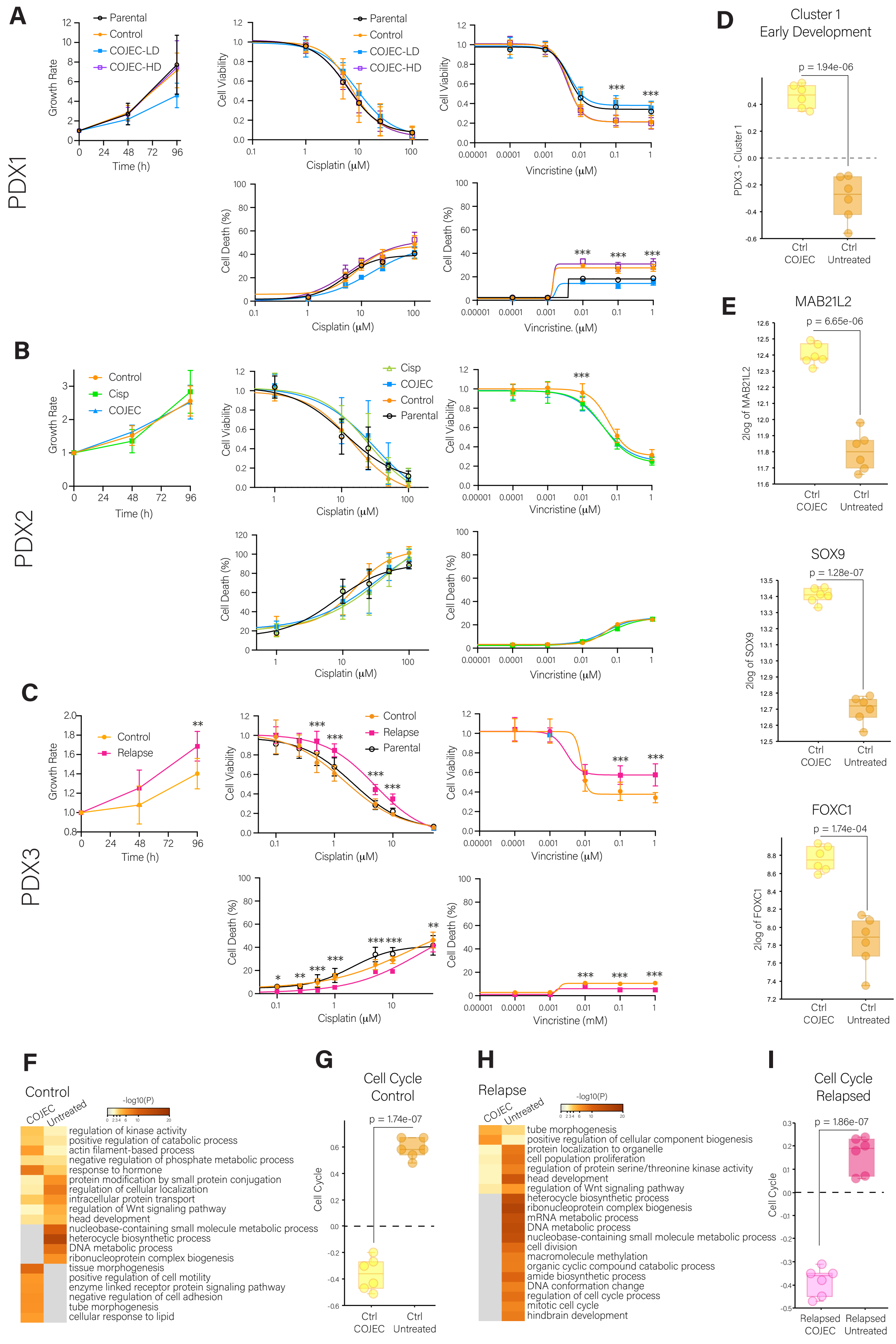

Fig. S10. Treatment and characterization of PDX-derived NB organoids. (**A-C**) Growth rate, cell viability, and cell death (%) for NB PDX-derived organoids treated with either Cisplatin or Vincristine as single drugs. Statistically significant differences were determined by two-way ANOVA with Šidák correction for multiple comparison (*, $p < 0.05$; **, $p < 0.01$, ***, $p < 0.001$). (**D, E**) Statistical analysis with Welch's t-test of Cluster 1 signature and expression of MES genes (*MAB21L2*, *SOX9*, *FOXC1*) with significantly different expression in COJEC-treated PDX3 control organoids compared to untreated organoids. (**F, H**) Gene ontologies defined by the top 1000 differentially expressed genes (DEG) between treatment groups for each set of NB organoids (Metascape enrichment analysis for GO Biological processes). (**G, I**) Statistical analysis with Welch's t-test of cell cycle gene expression between treatment groups for each set of NB organoids.

Supplementary Table 1

Treatment Response		
CR	Complete Response	Disappearance of tumor at least once
PR	Partial Response	Max Regression >50%
SD	Stable Disease	Max Regression <50%. Volume Increase at End of Treatment <25%
PD	Progressive Disease	Max Regression <50%. Volume Increase at End of Treatment >25%
- PD1	Aggressive Progression	TGD<=1.50
- PD2	Delayed Progression	TGD>1.50
TGD	Tumor growth delay	Survival day / Median survival day of controls

PDX1	Mouse Strain	Max regression (%)	Vol. Increase at End of Treatment (%)	TGD	Response	Surgery	Relapse
T1 (N)	Nude	0.00	209.37	1.60	PD2	No	
T2 (N)	Nude	0.00	169.11	1.00	PD1	No	
T3 - HD (N)	Nude	0.00	327.72	1.13	PD1	No	
T4 - HD (N)	Nude	0.00	278.86	2.87	PD2	No	
T5 - HD (N)	Nude	0.00	246.47	3.27	PD2	No	
T1	NSG	0.00	286.50	1.13	PD1	No	
T2	NSG	0.00	307.61	1.93	PD2	No	
T3	NSG	0.00	186.06	1.13	PD1	No	
T4	NSG	0.00	253.62	1.80	PD2	No	
T5	NSG	0.00	260.09	2.07	PD2	No	
T6	NSG	0.00	298.01	1.33	PD1	No	
T7	NSG	0.00	577.67	1.13	PD1	No	
T1 - HD	NSG	0.00	168.44	1.00	PD1	No	
T2 - HD	NSG	0.00	118.99	0.87	PD1	No	
T3 - HD	NSG	0.00	315.89	2.40	PD2	No	
T4 - HD	NSG	0.00	289.06	1.80	PD2	No	
T5 - HD	NSG	37.81	19.75		SD	No	
Cis 1	NSG	0.00	240.43	1.00	PD1	No	
Cis 2	NSG	0.00	301.11	1.13	PD1	No	
Cis 3	NSG	0.00	383.02	1.60	PD2	No	
Cis 4	NSG	0.00	222.58	1.47	PD1	No	
Cis 5	NSG	0.00	226.16	0.87	PD1	No	
**PDX2**	**Mouse Strain**	**Max regression (%)**	**Vol. Increase at End of Treatment (%)**	**TGD**	**Response**	**Surgery**	**Relapse**
T1	NSG	81.51	-59.07		PR	No	
T2	NSG	47.31	-8.16		SD	No	
T3	NSG	64.00	-64.00		PR	No	
T4	NSG	45.78	-24.94		SD	No	
T5	NSG	64.61	-40.14		PR	No	
T6	NSG	73.27	-53.18		PR	No	
T7	NSG	71.52	-71.52		PR	No	
T8	NSG	100.00	-100.00		CR	No	Yes
Cis 1	NSG	5.51	144.60	1.85	PD2	No	
Cis 2	NSG	57.70	-19.26		PR	No	
Cis 3	NSG	1.10	60.63	2.04	PD2	No	
Cis 4	NSG	14.26	-4.92		SD	No	
Cis 5	NSG	37.61	10.16		SD	No	
Cis 6	NSG	0.00	166.94	0.96	PD1	No	
Cis 7	NSG	30.36	35.96	2.31	PD2	No	
**PDX3**	**Mouse Strain**	**Max regression (%)**	**Vol. Increase at End of Treatment (%)**	**TGD**	**Response**	**Surgery**	**Relapse**
T1	Nude	75.63	-75.63		PR	Yes	Yes
T2	Nude	100.00	-48.40		CR	No	Yes
T3	Nude	72.13	-59.97		PR	No	
T4	Nude	67.19	-67.19		PR	Yes	No
T5	Nude	70.83	-70.82		PR	Yes	Yes
T6	Nude	55.04	2.63		PR	No	
T7	Nude	68.50	-61.79		PR	No	
T8	Nude	0.00	54.36	2.47	PD2	No	
T9	Nude	69.12	-69.12		PR	Yes	No
T10	Nude	20.95	-0.77		SD	No*	
T11	Nude	29.77	-3.26		SD	No	
T12	Nude	62.30	-62.30		PR	Yes	No
T13	Nude	61.63	-61.63		PR	Yes	No
T14	Nude	44.47	-44.47		SD	Yes	No
T15	Nude	63.83	-63.83		PR	Yes	No
T16	Nude	63.14	-63.14		PR	Yes	No
T17	Nude	100.00	-100.00		CR	No	Yes
Cis 1	Nude	39.84	31.65	3.46	PD2	No	
Cis 2	Nude	0.00	211.32	2.61	PD2	No	
Cis 3	Nude	23.07	276.37	2.38	PD2	No	
Cis 4	Nude	20.68	217.08	2.61	PD2	No	
Cis 5	Nude	23.58	11.21		SD	No	
Cis 6	Nude	20.16	-6.17		SD	No	
Cis 7	Nude	26.51	209.30	2.61	PD2	No	
Cis 8	Nude	27.34	48.96	3.31	PD2	No	
Cis 9	Nude	0.00	269.08	2.38	PD2	No	
Cis 10	Nude	0.00	317.82	1.54	PD2	No	

*Euthanized early due to weight loss. Included in the Surgey group due to collection time point.

Table S1. Individual PDX responses to COJEC or Cisplatin treatment. Detailed data of the individual response to treatment following the parameters of The pediatric preclinical testing program *(50)*. Max regression (%) represents the maximum tumor volume reduction during treatment time. The end of treatment was day 40 or the day of surgery. T – COJEC-treated mice, HD – high dose, Cis – Cisplatin treated mice, (N) – Nude mice.

Supplementary Table 2

Tumor groups based on COJEC treatment and response

PDX1	COJEC: Low Dose	COJEC: High Dose
T1 (N)	■	
T2 (N)	■	
T3 - HD (N)		■
T4 - HD (N)		■ D
T5 - HD (N)		■ D
T1	■	
T2	■	
T3	■	
T4	■	
T5	■	
T6	■	
T7	■	
T1 - HD		■
T2 - HD		■
T3 - HD		■ D
T4 - HD		■ scD
T5 - HD		■ D, scD

PDX2	COJEC	Relapse
T1	■	
T2	■	
T3	■ D	
T4	■	
T5	■ D	
T6	■	
T7	■ D	
T8		■

PDX3	Surgery Sample: Cured	Surgery Sample: Not Cured	Relapse	Regrow
T1		■ D, scD --->	■ D, scD	
T2			■ D, scD	
T3				■
T4	■ D, scD			
T5		■ D, scD --->	■ D, scD	
T6				■ D, scD
T7				■ D, scD
T8				■
T9	■ D, scD			
T10		■ *		
T11				■ D, scD
T12	■ D			
T13	■ D			
T14	■ D			
T15	■ D			
T16	■ D			
T17			■ D, scD	

*Euthanized early due to weight loss, so no paired relapse was available.

Table S2. Sample grouping based on COJEC treatment and response. Summary table showing the groups to which each sample was assigned based on treatment response, and the specific sequencing experiments performed. RNA sequencing was performed for all samples in this table. Bulk DNA sequencing was performed for samples marked as “D”. Single cell DNA sequencing was performed for samples marked as “scD”. Arrows indicate paired samples from the same mouse at different collection time points.

Supplementary Data Files

Data file S1. Genome-wide copy number profiles for all PDXs.

Data file S2. Complete lists of transcriptional signatures, DEG analysis and gene ontologies.

Data file S3. Complete data for the Metascape enrichment analysis and network plots.

Data file S4. Complementary overall survival Kaplan-Meier curves for various signatures.

Extended data: Bulk DNA copy number profiles.

PDX1

P1

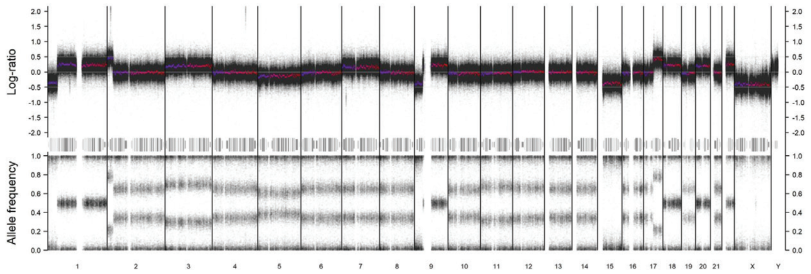

P2

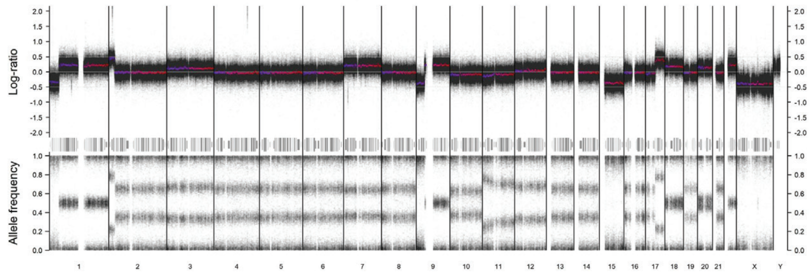

C1

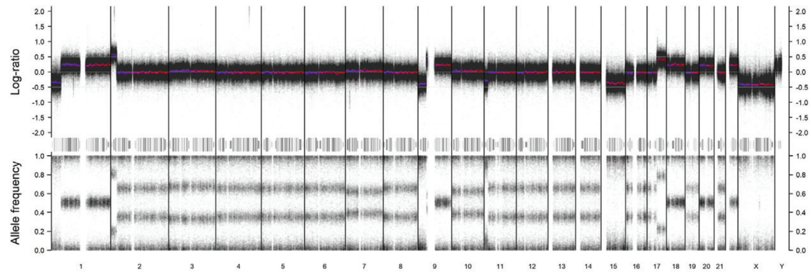

C3

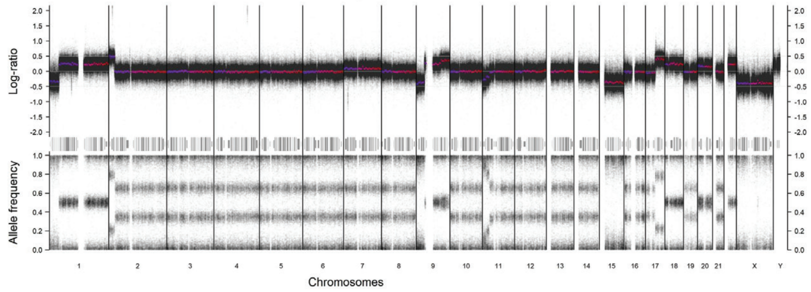

C5

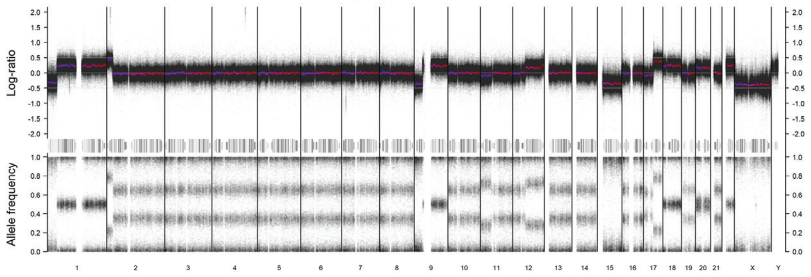

T4-HD (N)

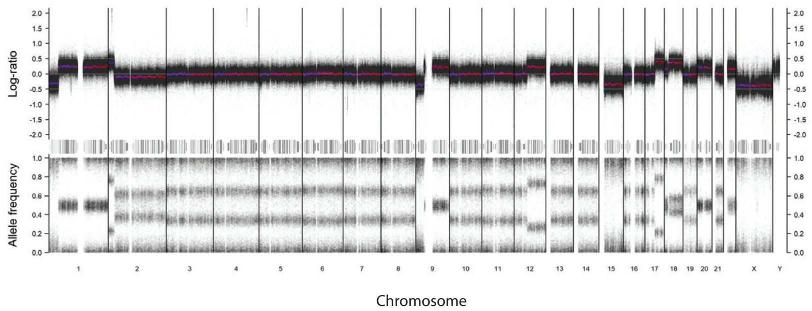

PDX1

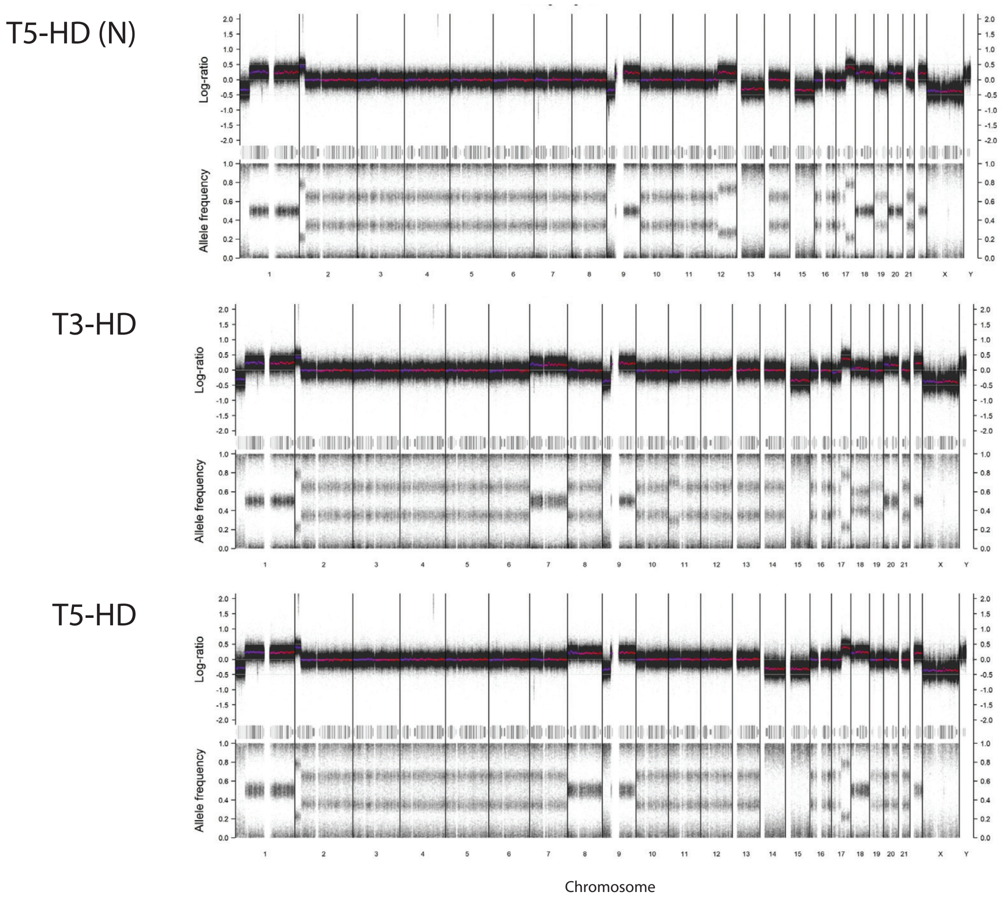

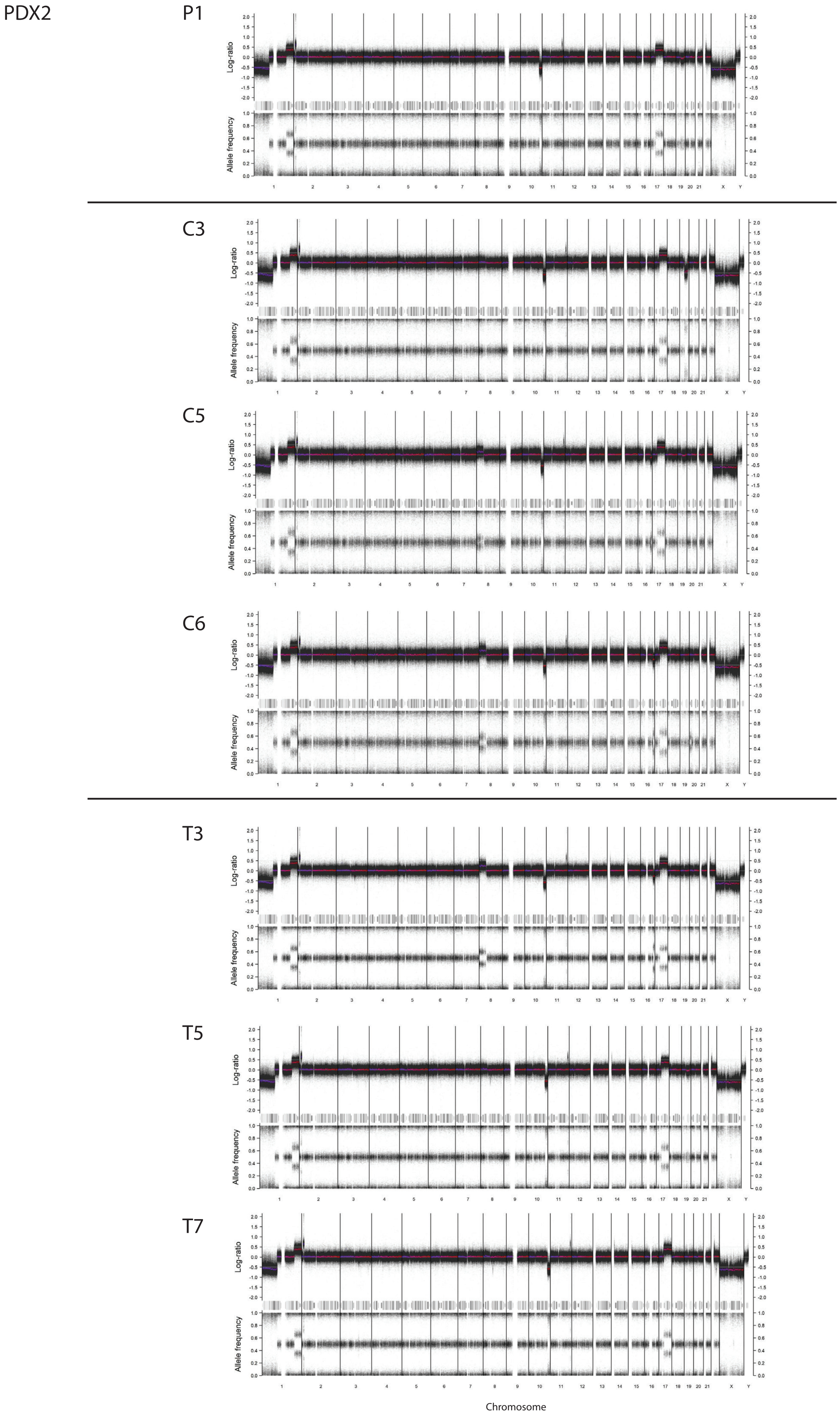
PDX2
P1
C3
C5
C6
T3
T5
T7
Log-ratio
Allele frequency
Chromosome
1 2 3 4 5 6 7 8 9 10 11 12 13 14 15 16 17 18 19 20 21 X Y

PDX3

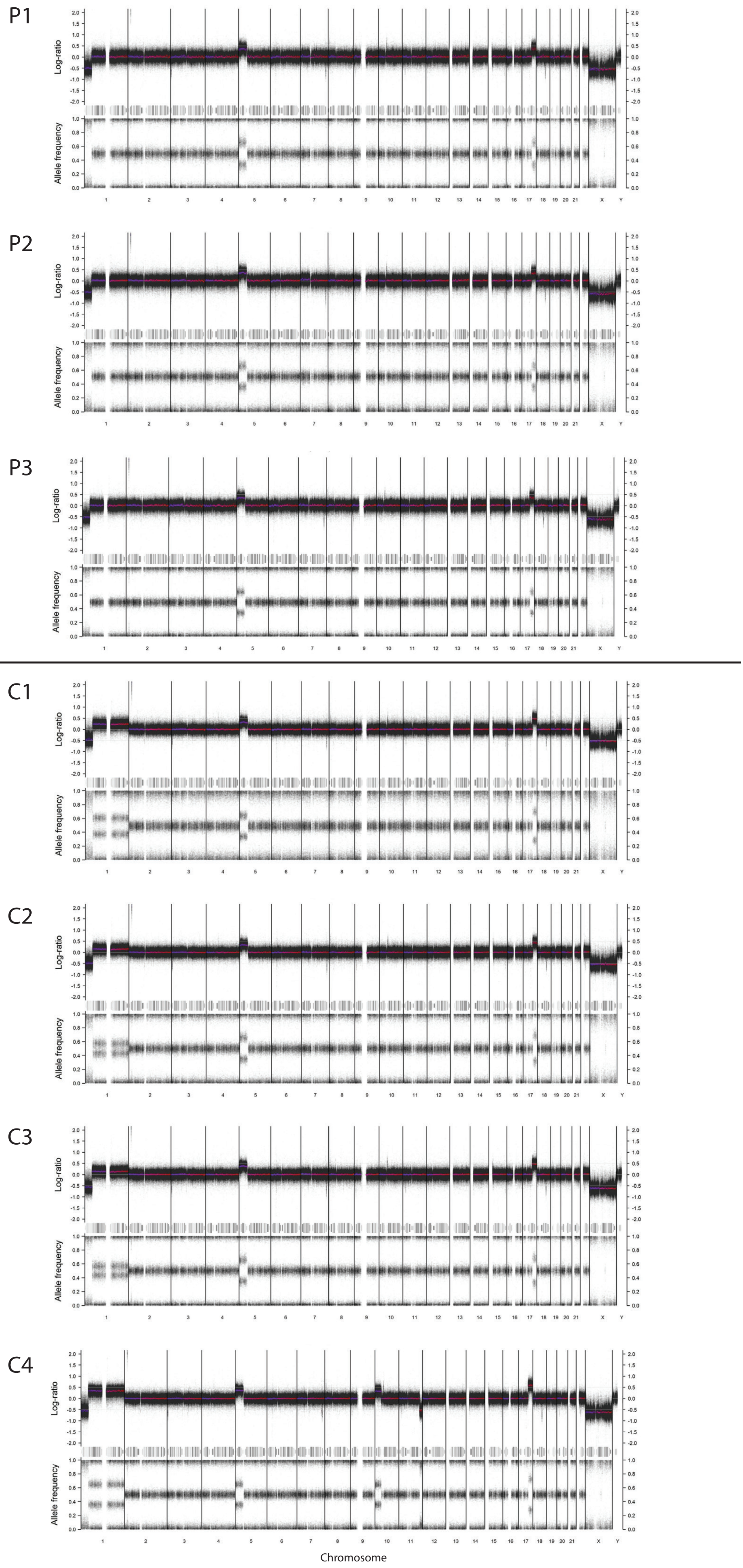
P1
P2
P3
C1
C2
C3
C4
Log-ratio
Allele frequency
1 2 3 4 5 6 7 8 9 10 11 12 13 14 15 16 17 18 19 20 21 X Y
Chromosome

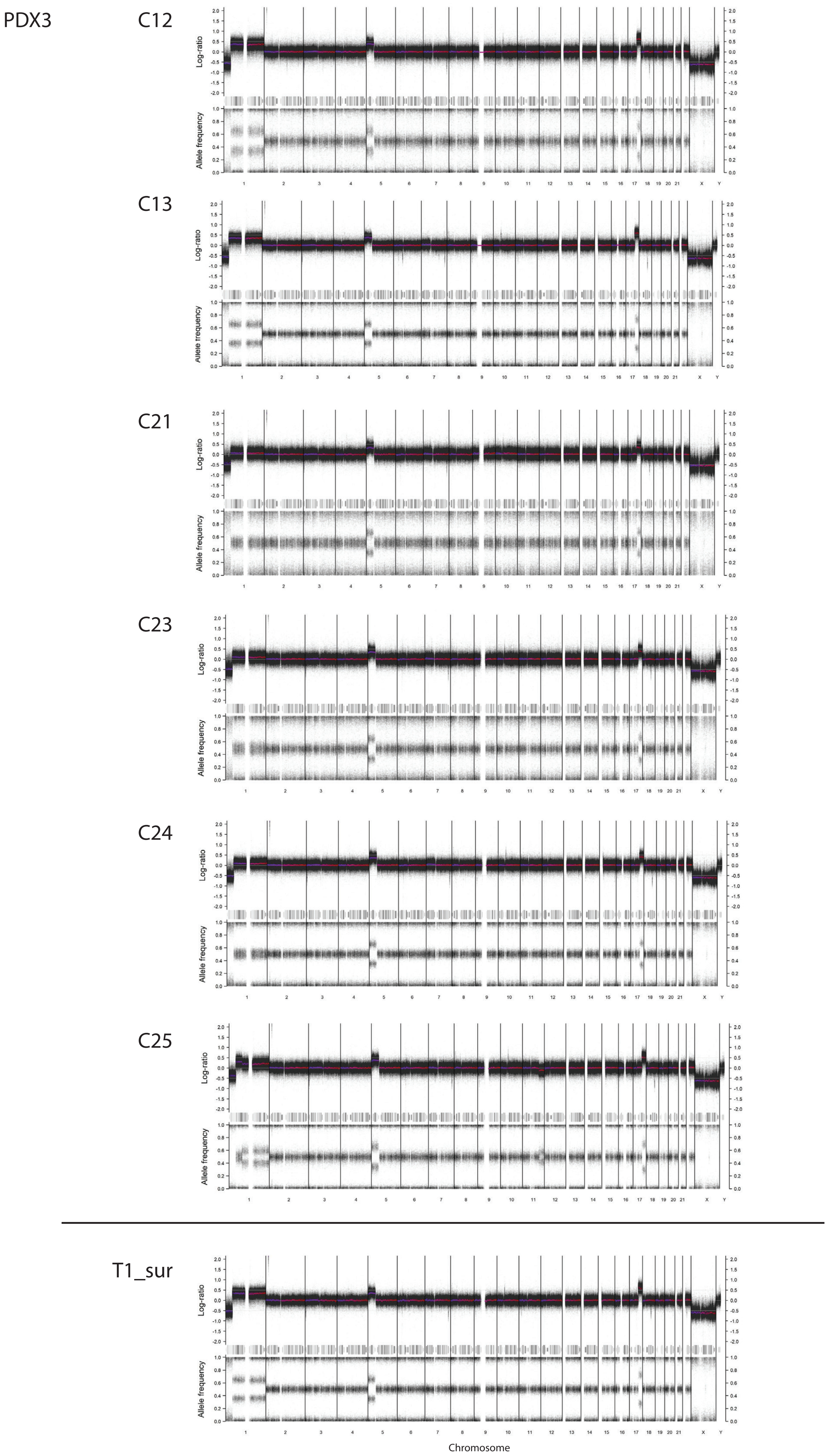
PDX3
C12
C13
C21
C23
C24
C25
T1_sur
Log-ratio
Allele frequency
Chromosome

PDX3

T4_sur

T5_sur

T9_sur

T12_sur

T13_sur

T14_sur

T15_sur

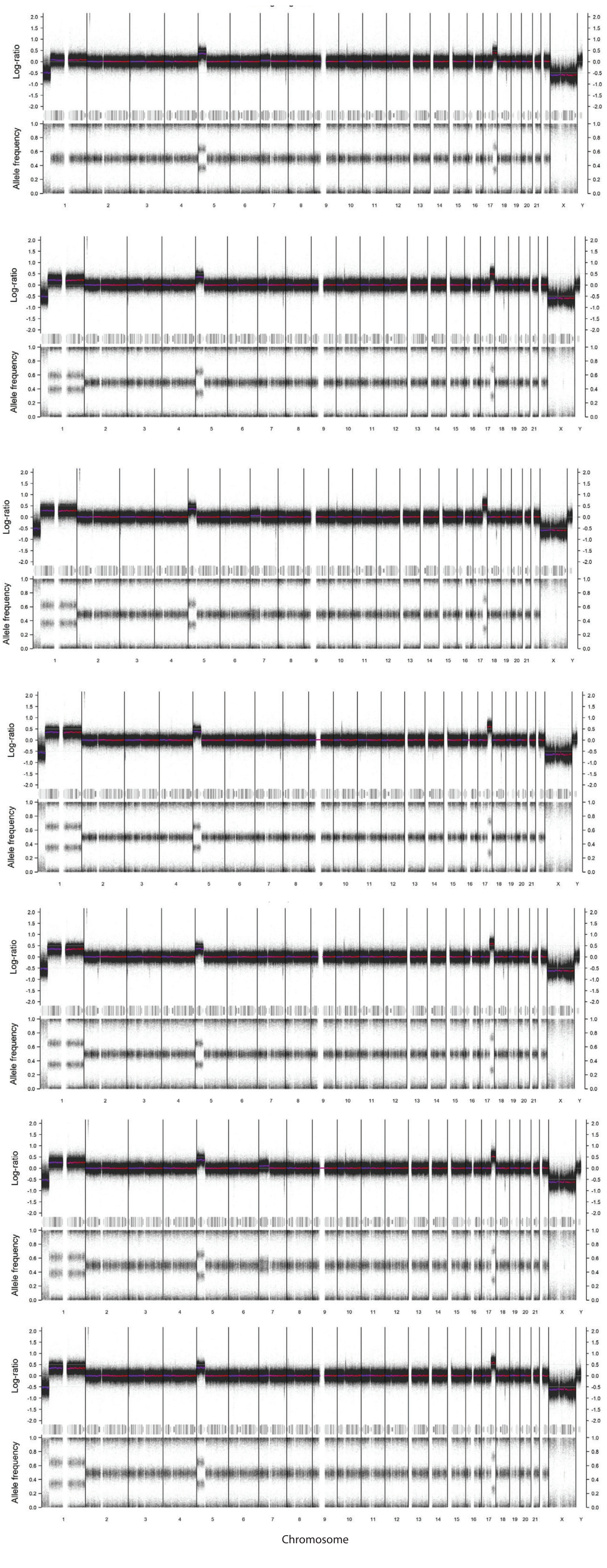

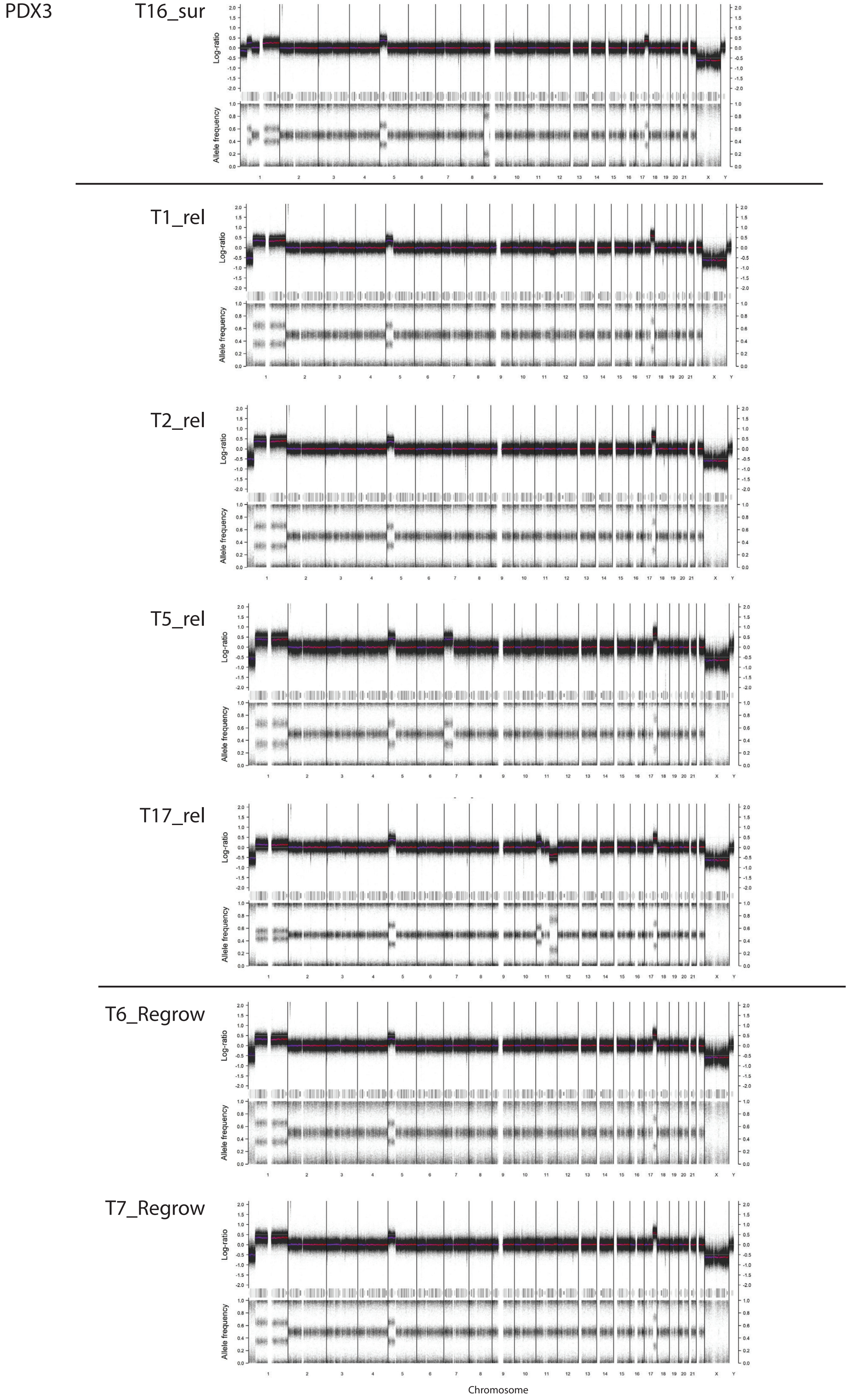
PDX3
T16_sur
T1_rel
T2_rel
T5_rel
T17_rel
T6_Regrow
T7_Regrow
Log-ratio
Allele frequency
Chromosome

PDX3

T11_Regrow

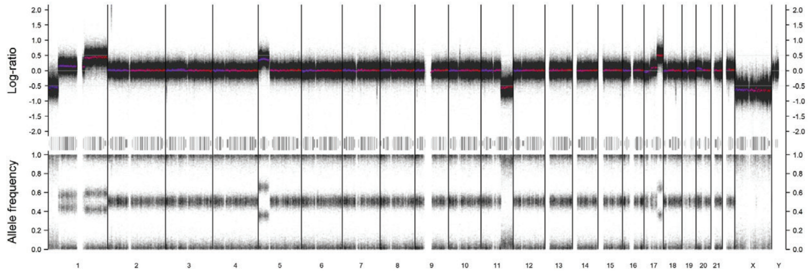

T5_Relapse_organoid

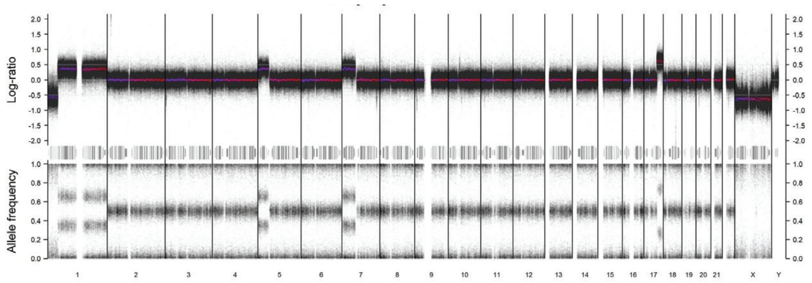

T11_Control_organoid

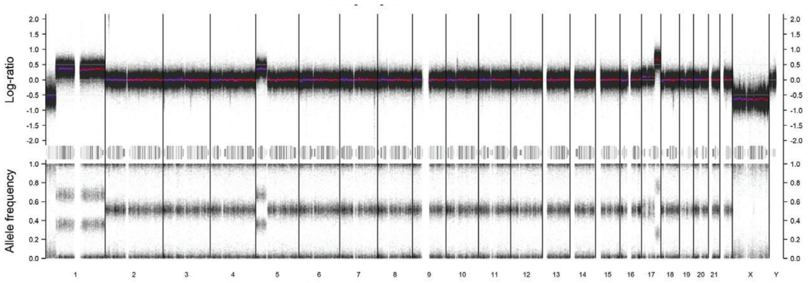

Chromosome

Suppplementary Data File S4

Survival for NB patients >18 months (Kocak Dataset) for the PDX3-derived transcriptional signatures

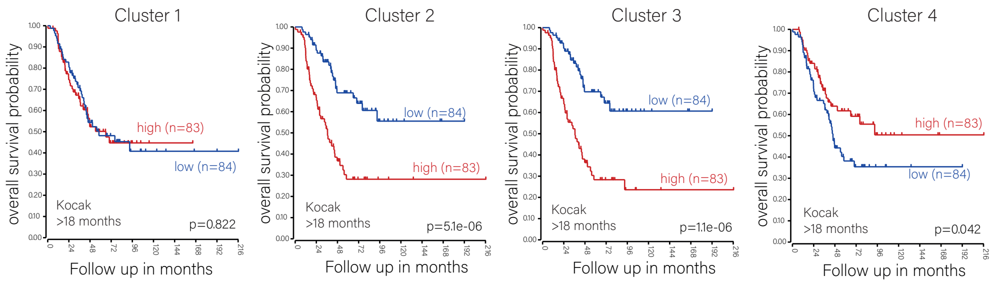

Survival for NB patients Stages 3-4 (Kocak Dataset) for the publicly available ADR/MES signatures

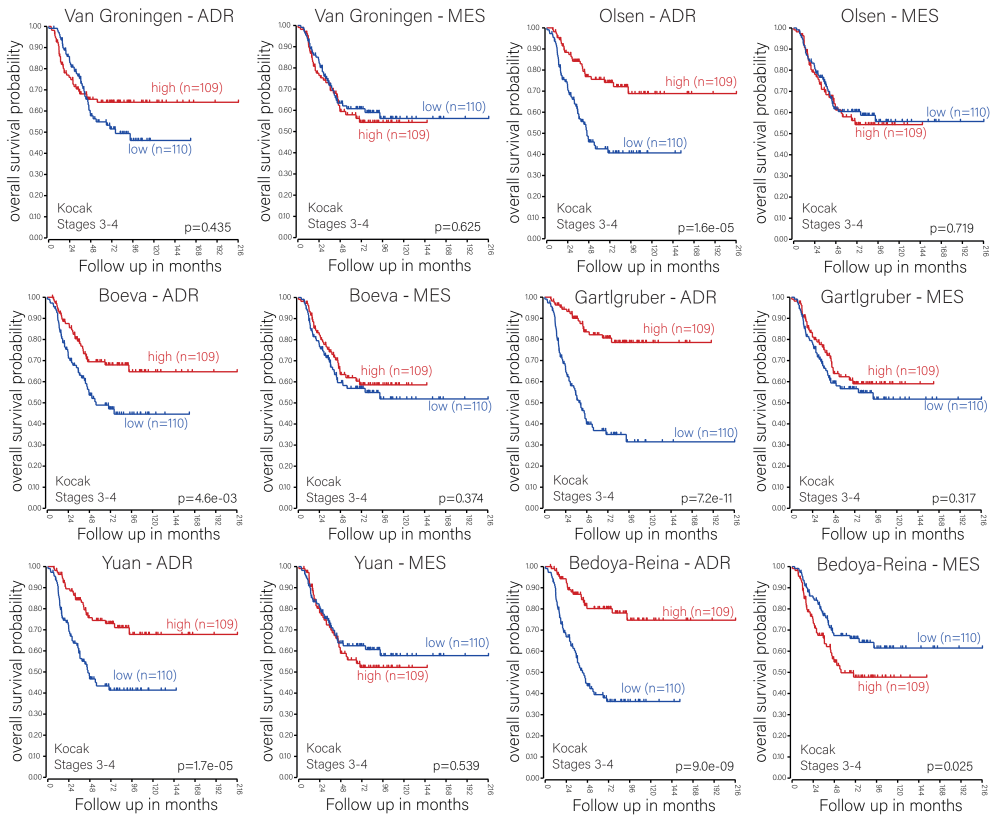

Survival for NB patients with *MYCN*-amp (Kocak Dataset) for the Merged and Integrated ADR/MES signatures

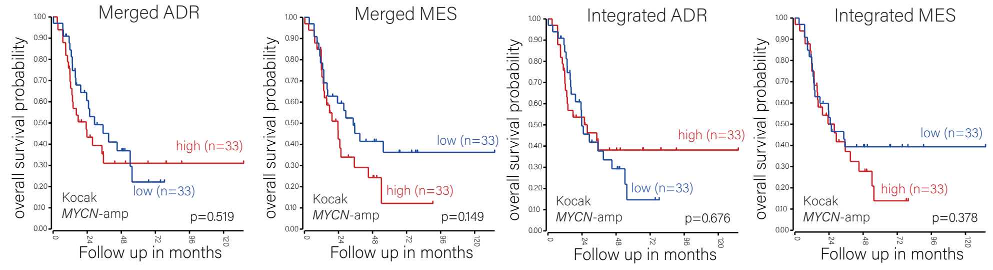

Survival for NB patients >18 months (Kocak Dataset) for the Merged and Integrated ADR/MES signatures

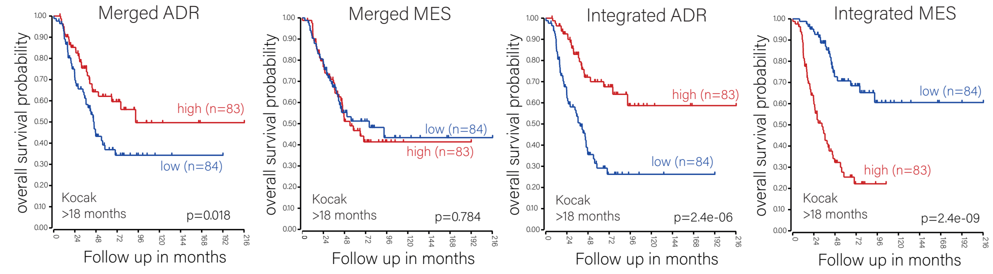

Supplement: Supplementary file 1 — Figs. S1 to S10 Tables S1 and S2 [file sciadv.abq4617_sm.pdf]
